# Preserved frontal lobe oxygenation following calcium chloride for treatment of anesthesia-induced hypotension

**DOI:** 10.3389/fphys.2014.00407

**Published:** 2014-10-22

**Authors:** Carl-Christian Kitchen, Peter Nissen, Niels H. Secher, Henning B. Nielsen

**Affiliations:** Department of Anesthesia, Rigshospitalet, University of CopenhagenCopenhagen, Denmark

**Keywords:** brain, blood pressure, cardiac output, NIRS, cerebral oxygenation, cerebral oximetry

## Abstract

Vasopressor agents may affect cerebral oxygenation (rScO_2_) as determined by near-infrared spectroscopy on the forehead. This case series evaluated the effect of calcium chloride vs. α and β-adrenergic receptor agonists on rScO_2_ in patients (*n* = 47) undergoing surgery during i.v. anesthesia. Mean arterial pressure (MAP) and cardiac output (CO) were assessed by Model-flow^®^ and ephedrine (55 ± 3 vs. 74 ± 9 mmHg; 10 mg, *n* = 9), phenylephrine (51 ± 5 vs. 78 ± 9 mmHg, 0.1 mg, *n* = 11), adrenaline (53 ± 3 vs. 72 ± 11 mmHg; 1–2 μg, *n* = 6), noradrenaline (53 ± 5 vs. 72 ± 12 mmHg; 2–4 μg, *n* = 11), and calcium chloride (49 ± 7 vs. 57 ± 16 mmHg; 5 mmol, *n* = 10) increased MAP (all *P* < 0.05). CO increased with ephedrine (4.3 ± 0.9 vs. 5.3 ± 1.2, *P* < 0.05) and adrenaline (4.7 ± 1.2 vs. 5.9 ± 1.1 l/min; *P* = 0.07) but was not significantly affected by phenylephrine (3.9 ± 0.7 vs. 3.6 ± 1.0 l/min), noradrenaline (3.8 ± 1.2 vs. 3.7 ± 0.7 l/min), or calcium chloride (4.0 ± 1.4 vs. 4.1 ± 1.5 l/min). Following administration of β-adrenergic agents and calcium chloride rScO_2_ was preserved while after administration of α-adrenergic drugs rScO_2_ was reduced by app. 2% (*P* < 0.05). Following α-adrenergic drugs to treat anesthesia-induced hypotension tissue oxygenation is reduced while the use of β-adrenergic agonists and calcium chloride preserve tissue oxygenation.

## Introduction

Cerebral autoregulation has a lower limit (Paulson et al., 1990) and following induction of anesthesia blood pressure may decrease to what is considered to be below that level. Accordingly, patients receive intravenous administration of vasopressor agents such as phenylephrine (an α-adrenergic receptor agonist) or ephedrine that stimulates both α- and β-adrenergic receptors. Bolus calcium chloride could also increase blood pressure (Ellender and Skinner, 2008) by an increase in intracellular calcium to increase cardiac stroke volume via an effect on myocytes and vascular resistance via increased contraction of smooth muscles. Also calcium chloride may increase venous return by unloading the splanchnic reservoir. Thus, with administration of calcium chloride cardiac output (CO) increases without affecting heart rate (HR) (Ellender and Skinner, 2008) contrasting ephedrine that has the potential to increase both HR and CO.

Phenylephrine decreases the near infrared spectroscopy (NIRS) determined frontal lobe oxygenation (rScO_2_) (Brassard et al., 2010, 2014; Nissen et al., 2010; Meng et al., 2011; Foss et al., 2014) related to vasoconstriction in extracranial vasculature rather than to a decrease in cerebral oxygenation (Ogoh et al., 2011, 2014; Sørensen et al., 2012). In this study patients undergoing major abdominal surgery were recruited to evaluate rScO_2_ following routine administration of vasoactive drugs to treat a drop in blood pressure by induction of anesthesia. We used bolus calcium chloride along with the vasopressor agents phenylephrine, ephedrine, adrenaline or noradrenaline depending on the choice of the anesthesiologist. We tested the hypothesis that administration of ephedrine, adrenaline and calcium chloride to treat anesthesia-induced hypotension would preserve rScO_2_, while rScO_2_ would be reduced following administration of drugs that stimulate α-adrenergic receptors (phenylephrine and noradrenaline).

## Methods

In a pilot-like prospective study-design as approved by the regional ethical committee (H-1-2009-107) we included predominantly patients planned for major abdominal surgery. In 47 patients (age 63 ± 7 yrs, height 176 ± 7 cm, weight 78 ± 16 kg; 28 males; mean ± SD) this selection of cases tested the effect of different vasopressor agents on anesthesia-induced hypotension and rScO_2_. Most patients were admitted for planned surgery including the liver, pancreas, esophagus, ventricle, or colon. In one case the spleen was the target for surgery and an other patient suffered from a retroperitoneal tumor. Three patients underwent vascular surgery and one patient was in surgery for hydronephrosis. Diabetes requiring insulin and the use of anti-hypertensive medication were considered to contradict inclusion in the evaluated series of patients. An increase in bilirubin was also an exclusion criterion due to the influence of bilirubin on near-infrared light absorption (Madsen et al., 2000).

The patients were exposed to at least 6 h of fast and orally intake of clear fluids was stopped 2 h before surgery. Three-lead electrocardiography monitored HR and pulse oximetry assessed arterial hemoglobin O_2_ saturation (SpO_2_). A hand vein was used for administration of fluid and anesthetics. According to local guidelines, a radial artery catheter (20 gage; 1.1 mm) was, after local anesthesia, inserted in the arm with the highest non-invasively determined systolic blood pressure and the catheter was kept patent by isotonic saline (3 ml/h) through to a transducer (Edwards Life Sciences, Irving, CA, USA) positioned at the level of the heart. For surgery an epidural catheter was placed at Th. 8–10 in the lateral decubitus position and under local anesthesia, lidocaine (3 ml, 10 mg/ml) with adrenaline (5 μg/ml) was administered to test for intravascular or intrathecal placement.

A two channel cerebral oximeter (INVOS 5100C, Somanetics, Troy, MI, USA) detected rScO_2_ that represents hemoglobin oxygen saturation in the tissue beneath the sensor as the ratio between oxygenated and total hemoglobin. As approved by the US Food and Drug Administration (510k-080769), the INVOS 5100C-determined rScO_2_ is considered a trend monitor of the hemoglobin O_2_ saturation for skin, scalp, and cortical tissue. With the NIRS-probe applied on the forehead it is assumed that capillaries within the frontal lobe contribute to light absorbance (Madsen and Secher, 1999) but skin, subcutaneous tissue and the scalp blood flow also influences the INVOS-determined rScO_2_ (Davie and Grocott, 2012). rScO_2_ was determined at least 2 cm above the eyebrows to limit an influence from the frontal sinus on rScO_2_ (Tubbs et al., 2002). Cardiovascular variables including mean arterial pressure (MAP), HR, cardiac stroke volume (SV) and thus CO were assessed invasively by Model-flow^®^ (Nexfin, B.V, Amsterdam, The Netherlands; Bogert and van Lieshout, 2005).

Anesthesia was induced with propofol (2 mg/kg) and maintained with propofol (0.08 mg/kg/min) and remifentanil (0.3–0.4 μg/kg/min). For ventilation a Dräger CATO (M32040, Lübeck, Germany) in volume-controlled mode was adjusted to an end-tidal CO_2_ tension of 4–4.5 kPa and a positive end-expiratory pressure of 5 cm H_2_O was used. When the patient was orally intubated, the inspiratory O_2_ fraction was set to 0.7 to preserve rScO_2_ (Rokamp et al., 2014). From induction of anesthesia, including tracheal intubation and until surgical incision, a reduction in MAP to below 60 mmHg was treated with administration calcium chloride (5 mmol) or α- and β adrenergic receptors: ephedrine (10 mg), phenylephrine (0.1–0.2 mg), adrenaline (1–2 μg), or noradrenaline (2–4 μg). No patient received more than one vasoactive agent.

It was estimated that to demonstrate a 10 ± 1.5% change in rScO_2_ (from 70% to 63%) as compared to the level before administration of the drug (alpha 0.05, power >90%) 17 patients was needed in each group. The goal was set to 20 patients to take drop-outs into account, but due to slow enrolment in study it was terminated. The study was conducted as open-label as the drug used depended on the choice of the anesthesiologist.

For comparison of values before and after drug administration a paired *t*-test (two tailed) was used and for comparison between groups and a *t*-test for unpaired data. For evaluation of differences in age, height and weight between groups we used ANOVA with unpaired data and a Tukey-test for *post-hoc* analysis. Analysis was performed by statistical software (PRISM 6.0 for MacOS; GraphPad software, San Diego, CA, USA) and a *P* < 0.05 was considered statistically significant.

## Results

Nine patients were provided with ephedrine to correct anesthesia-induced hypotension, phenylephrine was administered to eleven patients, noradrenaline to eleven patients, six patients received adrenaline, and calcium chloride was administered to ten patients. The five groups of patients were comparable in terms of height and weight but the patients in the adrenaline group were younger than those in the other groups (Table 1).

**Table 1:** **Patient characteristics in five groups of patients who received vasoactive therapy to treat anesthesia-induced hypotension**.

	**Ephedrine (*n* = 9)**	**Adrenaline (*n* = 6)**	**Phenylephrine (*n* = 11)**	**Noradrenaline (*n* = 11)**	**Calcium chloride (*n* = 10)**
Age (yrs)	67 ± 3	56 ± 11^*^	64 ± 7	64 ± 3	60 ± 9
Weight (kg)	84 ± 18	76 ± 13	76 ± 9	76 ± 16	80 ± 22
Height (cm)	178 ± 5	174 ± 6	179 ± 7	177 ± 7	172 ± 7

Variable are mean ± SD.

* Difference between Ephedrine and Adrenaline; P < 0.05.

**Figure 1:**
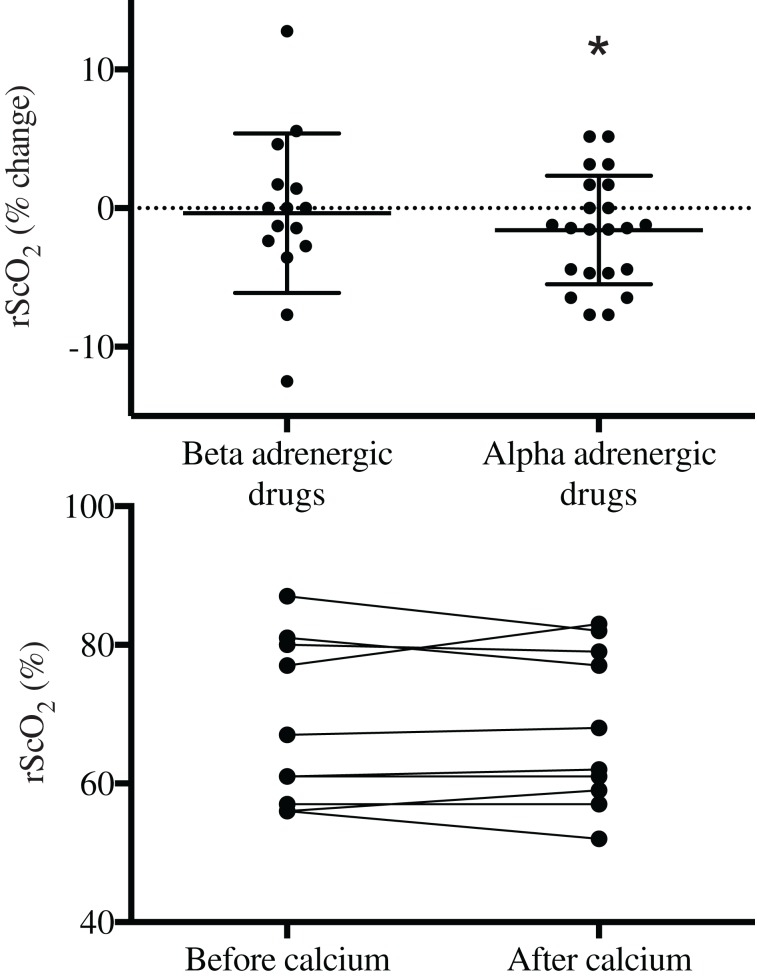
**Frontal lobe oxygenation (rScO_2_) in response to α- or β-adrenergic agents or to calcium chloride (lower panel) to treat anesthesia-induced hypotension in surgical patients**. ^*^Different from baseline; *P* < 0.05.

Bolus calcium chloride maintained HR, SV, and CO and, as intended MAP increased (from 49 ± 7 to 57 ± 16 mmHg, *P* < 0.05) (Table 2, Figure 1). The other vasoactive agents also influenced cardiovascular variables: following administration of adrenaline SV tended to increase (*P* = 0.08) and as HR was maintained (*P* = 0.71) also CO tended to increase (by 25%, *P* = 0.07). Similarly, administration of ephedrine increased CO (*P* < 0.05) due to a non-significant change in HR (*P* = 0.26) and SV (*P* = 0.10). Both adrenaline and ephedrine increased MAP by 19 mmHg. Phenylephrine and noradrenaline maintained HR, SV, and CO with an increase in MAP by 27 and 20 mmHg, respectively.

**Table 2:** **Cardiovascular variables in five groups of vasoactive therapy to treat anesthesia-induced hypotension**.

	**Adrenaline before**	**After**	**Ephedrine before**	**After**	**Noradrenaline before**	**After**	**Phenylephrine before**	**After**	**Calcium before**	**After**
HR (beat/min)	65 ± 5	63 ± 9	60 ± 9	64 ± 18	57 ± 18	58 ± 19	56 ± 11	55 ± 12	61 ± 14	58 ± 15
SV (ml)	72 ± 17	89 ± 10^*^	76 ± 14	82 ± 15^*^	64 ± 14	70 ± 12	66 ± 9	67 ± 11	65 ± 21	67 ± 17
CO (L/min)	4.7 ± 1.1	5.9 ± 1.1^*^	4.3 ± 0.9	5.3 ± 1.2^*^	3.8 ± 1.2	3.7 ± 0.7	3.9 ± 0.7	3.6 ± 1.0	4.0 ± 1.4	4.1 ± 1.5
MAP (mmHg)	53 ± 3	72 ± 11^*^	55 ± 3	74 ± 9^*^	53 ± 5	72 ± 12^*^	51 ± 5	78 ± 9^*^	49 ± 7	57 ± 16^*^
rScO_2_ (%)	58 ± 13	58 ± 12	73 ± 10	73 ± 11	70 ± 12	68 ± 11	67 ± 8	66 ± 7	68 ± 12	68 ± 11

Variables are mean ± SD. HR, heart rate; SV, cardiac stroke volume; CO, cardiac output; MAP, mean arterial pressure; rScO_2_, frontal lobe oxygenation.

* Different between before and after in each group; P < 0.05. In calcium group determination of CO and SV was successful in six patients. After adrenaline SV and CO changed with P-values at 0.08 and 0.07, respectively. Epedrine affected SV at P = 0.10.

**Figure 2:**
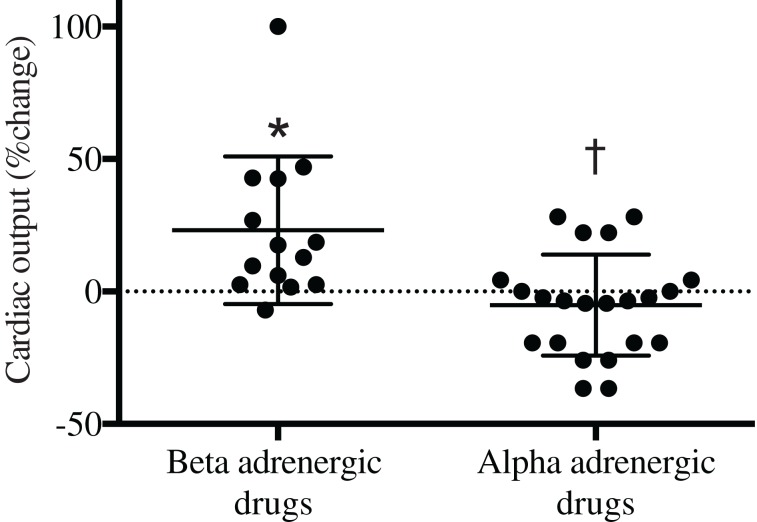
**Effects of α- or β-adrenergic agents on cardiac output following anesthesia in surgical patients**. ^*^Different from baseline; †, difference between groups. *P* < 0.05.

The effect of vasoactive therapy on the NIRS determined rScO_2_ are shown in Table 2 and Figure 1. In patients treated with adrenaline and ephedrine rScO_2_ was not affected significantly and when these data were pooled into one group of patients treated with β-adrenergic drugs, rScO_2_ remained statistical unaffected: there was an increase in rScO_2_ for five patients and for seven patients rScO_2_ decreased without relation to changes in MAP or CO. After noradrenaline and phenylephrine a small but non-significant reduction in rScO_2_ was noted for each vasoactive agent. However, with data evaluated as one group (α-adrenergic receptor agonists; noradrenaline and phenylephrine), seven patients with noradrenaline and seven patients with phenylephrine demonstrated lowered rScO_2_ after drug administration while for only six patients rScO_2_ increased (Figure 2). For two of these patients rScO_2_ decreased almost 10% while CO increased (0.5 and 2.3 L/min) and for the whole group of patients (α-adrenergic receptor agonists) rScO_2_ decreased 2%. After calcium chloride in four patients rScO_2_ decreased (1–5%) and while rScO_2_ was unchanged (*n* = 2) or increased (up to 6%) in the other eight patients, rScO_2_ was not statistical affected by calcium chloride. Correlations between rScO_2_ and MAP or CO were not observed.

## Discussion

This case series of 47 patients confirms that following anesthesia-induced hypotension in elective surgical patients, a vasopressor agent including calcium chloride increases MAP. The new finding is that frontal lobe oxygenation (rScO_2_), as determined by near-infrared spectroscopy was not significantly affected following the use of calcium chloride for treatment of anesthesia-induced hypotension. A similar finding was observed with the use of ephedrine, phenylephrine, adrenaline, and noradrenaline as rScO_2_ remained at levels similar to those established before drug administration. However, when data from patients treated with α-adrenergic receptor agonists (phenylephrine and noradrenaline) were pooled into one group and patients treated with β-adrenergic drugs (adrenaline or ephedrine) were sampled in an other group, rScO_2_ decreased 2% after α-adrenergic drug administration but remained unaffected with administration of β-adrenergic stimulating drugs. This observation supports results obtained in patients (Nissen et al., 2010; Meng et al., 2011; Brassard et al., 2014) and healthy awake subjects (Brassard et al., 2010). Although a 2% reduction in rScO_2_ seems small, the change is in the magnitude as induced by hyperventilation that lowers arterial CO_2_ partial pressure with development of presyncopal symptoms (Madsen and Secher, 1999). Also Thomas et al. (2009) report ~6% drop in cerebral oxygenation at presyncope.

Why rScO_2_ is reduced after α-adrenergic drugs and not after administration of β-adrenergic-therapy and calcium chloride remains unclear. In patients with intact cerebral autoregulation, the decrease in rScO_2_ after phenylephrine and noradrenalin administration is associated with concordant reduction in CO, whereas rScO_2_ remains unchanged when CO was maintained with ephedrine (Meng et al., 2011). This observation supports that changes in CO, independently of arterial pressure, affect cerebral hemodynamics (Ogoh et al., 2005). Cerebral arteries are abundantly innervated by sympathetic fibers (Sandor, 1999) and the decrease in rScO_2_ after administration of α-adrenergic drugs could be by direct α-receptor-mediated cerebral vasoconstriction. An influence of cutaneous vasoconstriction beneath the NIRS optode, however has to be considered (Davie and Grocott, 2012; Sørensen et al., 2012).

The increase in MAP by vasoactive therapy was expected and the use of bolus calcium chloride also increased MAP even in patients without a suspected reduction in plasma ionized calcium. Indication for the use of calcium chloride is more clear after the use of blood products containing citrate that may lead to hypocalcemia (Jawan et al., 2003) and hemodynamic instability (Marquez et al., 1986). Thus, calcium chloride restores the levels of ionized calcium in blood and in turn also MAP.

Despite the effect of vasoactive agents on MAP was similar with the use of different drugs, the rise in pressure was achieved differently. Both ephedrine and adrenaline increased CO but such an effect was absent after administration of phenylephrine or noradrenaline. When data from patients treated with noradrenaline and phenylephrine were pooled into one group, CO was reduced in several patients (Figure 2): the eight lowest points with a decrease in CO by up to app. 50% represent four patients treated with phenylephrine and four patients treated with noradrenaline. After calcium chloride CO was unchanged and there was a trend towards a reduced HR (*P* = 0.16). For most of the patients treated with β-adrenergic agonists, CO increases by unloading of the splanchnic reservoir (Cannesson et al., 2012). An increase in CO may be important to override potential vasoconstriction in cutaneous and subcutaneous vessels following the use of vasoactive therapy. Considering that microvascular circulation influences wound healing, we consider that vasoactive drugs with significant vasoconstrictive capacity should be avoided both during and after surgery.

The use of calcium chloride to restore MAP is safe and in terms of the effect on rScO_2_ this study is unable to promote one vasoactive drug over an other. The data do suggest that the use of a vasopressor agent with combined α- and β-adrenergic agonistic capacity appears to be favorable to restore MAP following anesthesia-induced hypotension. Results from a randomized double-blinded clinical trial are needed before a general recommendation to use combined α- and β-adrenergic drugs or calcium chloride for treatment of anesthesia-induced hypotension is substantiated.

### Conflict of interest statement

The authors declare that the research was conducted in the absence of any commercial or financial relationships that could be construed as a potential conflict of interest.
